# Acetabular revision surgery revisited: do changing indications influence bone defect management? A comprehensive review

**DOI:** 10.1530/EOR-2026-0029

**Published:** 2026-10-01

**Authors:** Eduardo García-Rey, Sebastien Lustig, Theofilos Karachalios, Luigi Zagra

**Affiliations:** ^1^ Department of Orthopedic and Traumatology, La Paz University Hospital-IdiPaz, Madrid, Spain; ^2^ Orthopaedics Surgery and Sports Medicine Department FIFA Medical Centre of Excellence, Croix-Rousse Hospital Lyon University Hospital Lyon France, Lyon, France; ^3^ Department of Orthopaedic Surgery and Musculoskeletal Trauma, University General Hospital of Larissa, School of Health Sciences, Faculty of Medicine, University of Thessaly, Larissa, Thessalia Sterea Ellada, Greece; ^4^ Hip Department, IRCCS Istituto Ortopedico Galeazzi, Milan, Italy; ^5^ Università Vita-Salute San Raffaele, Milan, Italy

**Keywords:** revision hip surgery, acetabular bone defects, bone graft, reconstruction hip implants

## Abstract

- Modern bearings have changed reasons for revision hip surgery; however, acetabular bone defects are still a challenging condition.
- Advanced imaging techniques, including newer 3D preoperative planning, have improved the understanding of bone stock.
- Current acetabular tools reduce the bone loss during implant removal and are reliable to predict the new reconstruction.
- Although bone allograft is useful in younger patients with a high lifetime risk for revision, some limitations have been reported for segmental large defects and pelvic discontinuity.
- Encouraging results are obtained with porous metal augments and custom-made implants in combination or not with bone grafts, but long-term results and cost-effectiveness are controversial.
- In this review, we aimed to address the best rationale to this complex topic 25 years after the introduction of highly cross-linked polyethylene. Furthermore, we sought to identify whether the lower prevalence of osteolysis eases acetabular revision surgery within the increasing number of re-revision surgeries.

## Introduction

Acetabular side has been the weak point since the introduction of modern total hip arthroplasty (THA). The excellent results for low-friction arthroplasty for the Charnley femoral component were not as satisfactory for the acetabular one (1). Complex acetabular pathologies, such as congenital hip disease, rheumatologic conditions or post-traumatic acetabula, were the most frequent reasons for early revision surgery due to failure of fixation of the acetabular component, whereas polyethylene (PE) wear at a long term was the reason for late revisions (2). Moreover, osteolytic lesions may develop from a biological reaction to wear debris, most frequently polyethylene and metal debris (3). Although uncemented fixation showed promising results on bone osseointegration, when these components were used in combination with ‘old’ PE gamma-irradiated in air, there were increasing revision rates due to wear, osteolysis and aseptic loosening secondary to oxidation and degradation of the liner (4). After the widespread use of highly cross-linked PE (HXLPE) during the past two decades, the reasons for revision hip surgery dramatically changed (5). Prospective comparative clinical and radiological investigations at a long term and national registries assessing modern bearings confirmed the early results found in the laboratory (6, 7). As a consequence of these results, the use of modern wear-resistant bearings, including HXLPE and ceramic-on-ceramic bearings, increased all around the world with significant difference among countries (8).

Despite all these developments in THA technology, revision surgery rates are increasing worldwide. The Annual Report 2024 of the Swedish Arthroplasty Register described that loosening was the most frequent revision since 2003; however, its relative proportion has gradually decreased (9). During the past years, infection is gaining on the proportion of revisions, patients’ age is increasing and other challenging conditions such as having more diagnoses other than osteoarthritis and comorbidities are more frequent than in primary THA. Furthermore, although long-term results for primary and first-time revisions are improving, the risk of re-revision for infection, with worse outcomes than revision for other reasons, is concerning. Similarly, other relevant issues, such as metal-on-metal articulations or corrosion complications, increased the number of THA failures and the complexity of acetabular revision surgery (8). Consequently, although osteolysis is less frequent nowadays, acetabular bone loss is always to be noted regardless of the cause for revision.

In this comprehensive review, we aimed to identify whether current indications for acetabular revision surgery affect the hip reconstruction. Hence, we sought to assess modern surgical knowledge before, during and after revision THA with particular attention to acetabular bone defect.

## Preoperative assessment in acetabular revision surgery

### Identifying the previous implant

After evaluating the patient, including the reason for revision, the surgeon is ready to start the preoperative planning. As mentioned above, osteolysis rates are decreasing during the past years after THA; however, infection, dislocation and periprosthetic fractures frequently need the removal of the acetabular component, which is frequently fixed to the bone. First, the implant type must be recognized. The so-called ingrowth cups have a porous-coated interface leading to osseointegration and increasing difficulties during the removal surgery since more bone loss can occur than expected (10). On the contrary, removal of ongrowth implants is associated with minimal difficulties with current tools (Fig. 1). The identification of screws, liner locking mechanism type, or a cemented one, in the previous acetabular component is also essential when planning surgery (11). Finally, corrosion or other adverse reactions to metallic debris due to backside wear, fretting from screws or metal-bearings can be present, leading to bone loss and soft tissue damage (12, 13).

**Figure 1:**
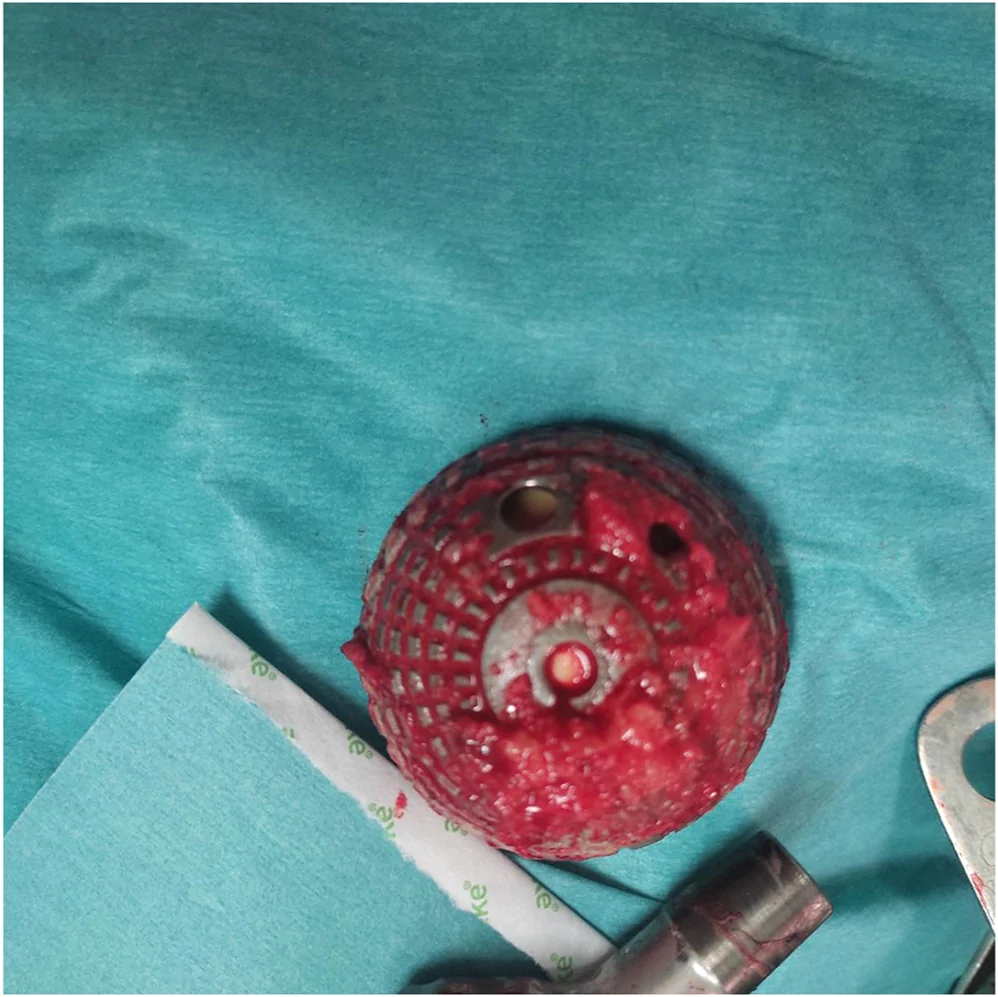
Uncemented acetabular component removed with a modern tool. Minimal bone loss can be noted.

### Classifying acetabular bone defect

Many classifications for acetabular bone defects were developed. They are useful in planning the surgery, particularly those suggesting treatment based on the extent of bone loss and the type of defect, for example segmental/uncontained or cavitary/contained (14, 15). The Paprosky classification is worldwide recognized to determine the surgical management as reported by Telleria and Gee (16). In their article, they emphasized the importance of the supporting structures of the acetabulum as observed on AP radiographs of the pelvis to visualize the teardrop, migration of the hip centre, the Kohler line and the presence or absence of ischial lysis. Additionally, the authors highlighted its widespread use, simplicity and reliability. Nevertheless, all classifications have some limitations, such as difficulties when assessing osteolysis, and modern imaging provides more interesting observations. The international consensus is trying to include recent knowledge as in other challenging topics (17). Despite all these previously reported methods to improve surgical management, it is essential to highlight that the intraoperative bone defect after removing the acetabular component, as previously described, will always determine the treatment and the prognosis. After the introduction of uncemented acetabular components showing excellent bone fixation, excessive bone loss was frequently associated after cup removal (18).

Conventional radiographs are critical to suspect loosening, osteolysis or other changes around the acetabular component. Moreover, anteroposterior pelvic views are very useful to diagnose pelvic discontinuity; asymmetry between both foramen obturator and Judet views are meaningful to identify damage on the anterior and posterior columns; however, modern computed tomography (CT) imaging outperforms simple radiographs in most cases (19). Osteolysis, screw placement, modular liner damage, corrosion or joint fluids can be detected more easily with CT scans (Fig. 2). At present, 3D reconstruction is able to clearly define the limits of a strong area to support a three-point fixation with an uncemented conventional acetabular component. Also, the quadrant technique for ideal screw positioning has been simplified using these 3D models, in particular for ischiatic fixation.

**Figure 2:**
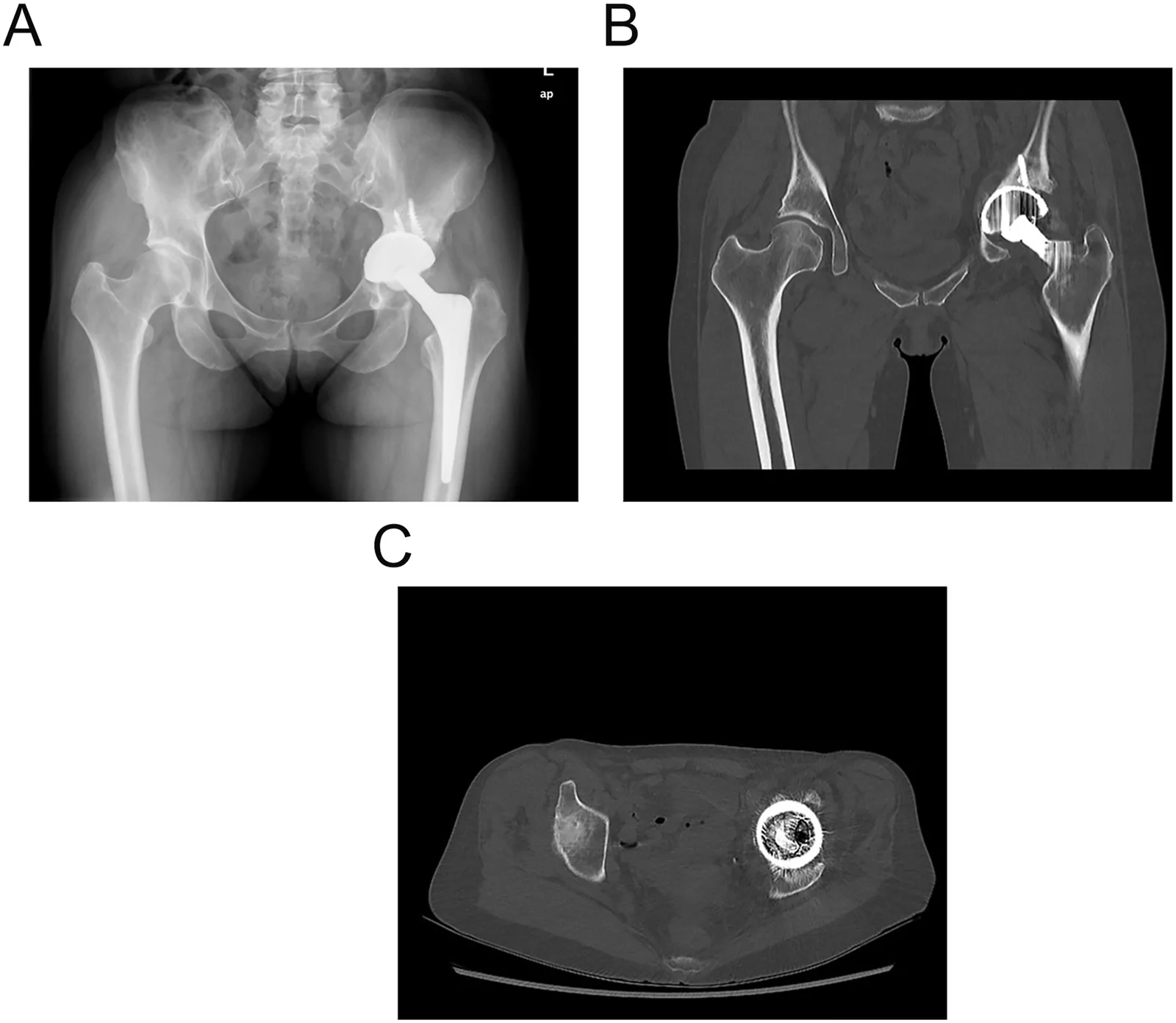
(A) Anteroposterior radiograph of a 50-year-old patient showing a periprosthetic acetabular fracture after a fall. (B) Coronal view on a computed tomography (CT). (C) Axial CT view.

Current 3D technology can create a biomodel from CT imaging to improve acetabular revision preoperative planning (20). Multidisciplinary collaboration between surgeons and engineers is essential to obtain the potential benefits of this technique. Pelvic bone anatomy is challenging during revision surgery. Furthermore, previous implants can affect the quality of the CT imaging and make differentiating between bone and the metallic structures within the bone difficult (Fig. 3).

**Figure 3:**
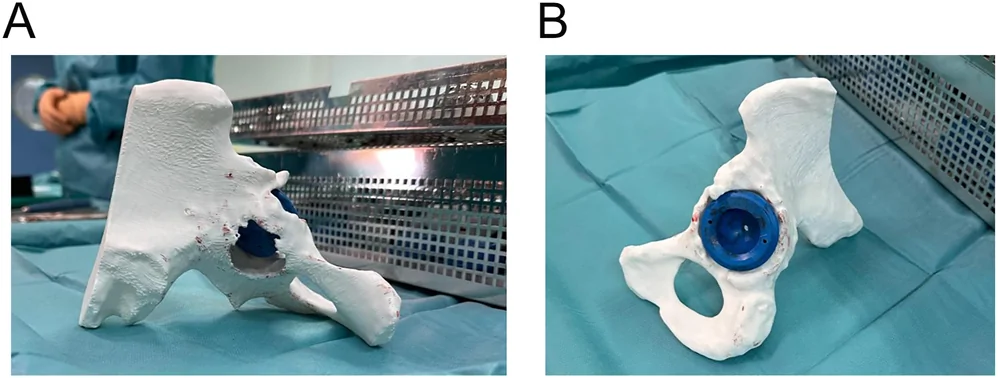
(A) Intraoperative view of the 3D biomodel of the same clinical case. (B) Trial acetabular component onto the 3D biomodel.

After identifying all these possible artefacts, the bone defect can be defined. Despite these advantages, the surgeons must be focused on the possibility of bone loss enlargement after implant removal. At present, the number of previous surgeries and the aetiology of the pathology leading to primary THA also play a critical role in the success of the procedure. Altering anatomy after pelvic osteotomy, metal hardware or osteotomies around the proximal femur influence the surgical approach, soft tissue damage or scarring (21). The surgeons must be aware of the unexpected events and associated causes during revision surgeries and the acetabular revision surgery armamentarium.

## Reconstruction options for acetabular revision surgery

### Bone allograft reconstruction

In most cases with small or moderate acetabular bone defects, a conventional cup with a highly porous surface can be implanted (22). However, in some cases, the surgeon must decide whether to remove a well-fixed porous-coated cup when reoperating for osteolysis and polyethylene wear. Although this is an infrequent clinical situation nowadays, Maloney *et al.* pointed out the possibility of performing bone grafting in a well-fixed cup if the position is acceptable and the PE liner is replaceable (23).

After the removal of the previous acetabular component using modern explant tools, which minimizes bone loss during surgery, the intraoperative defect will be finally defined (24). If the bone loss is greater than 50%, although some studies now accept 30% of host bone contact and the trial of a hemispherical component is not enough to achieve a three-point fixation around the proximal-ilium and distal-pubis and ischium, then an alternative must be undertaken (25, 26). Although the reconstruction device is selected by the surgeon according to his/her expertise, bone allograft is going to be used frequently.

Bulk bone allograft was a common indication; however, the problems found with regard to resorption in the weight-bearing area of the acetabulum, particularly when these grafts were not protected by a cage, limited its use (27). A recent report by Sherson *et al.* confirmed all these recommendations (28). Similarly, with some particular reconstruction rings or cages, such as the Kerboull Cross, combined with this type of bone allograft, acceptable results are reported (29). In their study, Gibon *et al.* analysed 37 hips with Paprosky III defects, finding a 10-year survival rate of 95.3% for aseptic loosening and 76.5% for radiological loosening, which resulted in a reasonable option for these complex cases.

The benefits of using bone allograft in moderate and large acetabular defects in revision hip surgery are well known. Studies assessing hip reconstruction confirmed that it is possible to recreate a more anatomical situation. The most validated technique using bone allograft is bone impaction grafting. Developed during the early 1980s for acetabular protrusion in primary THA using the autograft from the femoral head, it was used in revision THA (30). Preparing the graft, morselizing by hand using a rongeur and vigorous impaction with hemispherical instruments after containing the segmental defect are key steps (Fig. 4). The use of fresh frozen allografts from local or regional banks is recommended for both techniques (31). Although first described for cemented cups, this technique can be used also with uncemented acetabular components or other reconstruction devices (32) (Fig. 5). To date, smaller bone defects when a second revision has been required than the previous one were found, confirming the benefits of bone stock restoration (33). Recently, more studies from different countries are providing evidence of the advantages of this technique when used in combination with modern cages in severe bone defects, including pelvic discontinuity, and when using a cemented cup and metal-mesh containment at a very long-term follow-up (34, 35)

**Figure 4:**
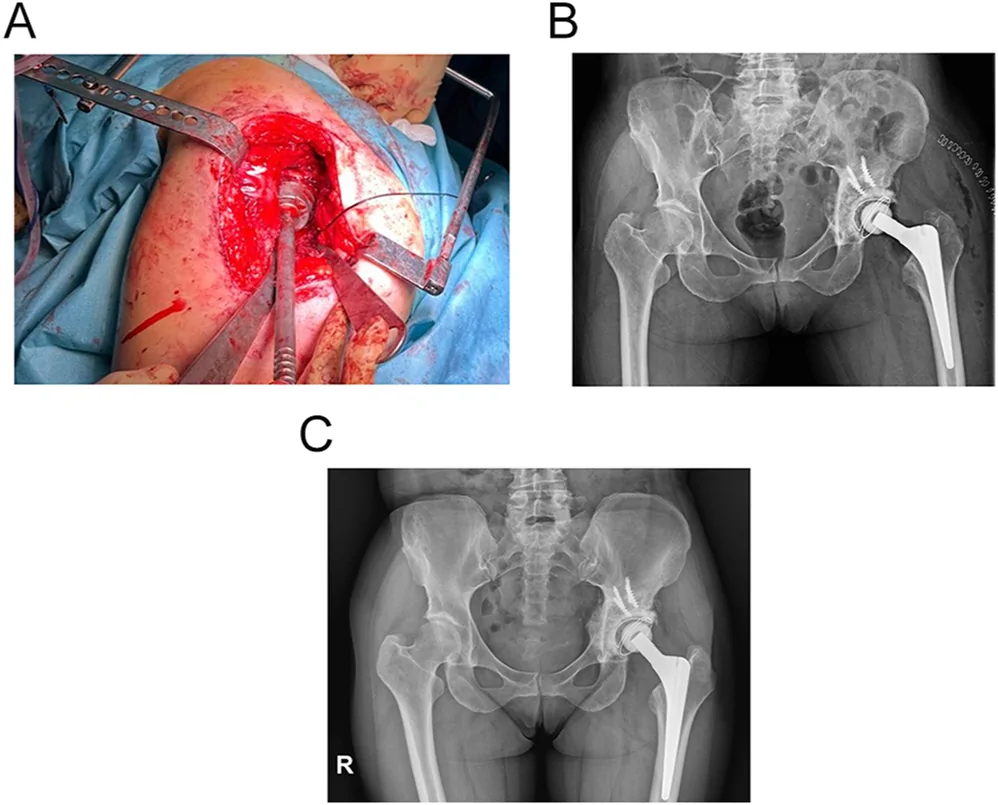
(A) Acetabular bone impaction allografting using a cemented cup of the same clinical case. (B) Six-week postoperative radiograph. (C) One-year postoperative radiograph.

**Figure 5:**
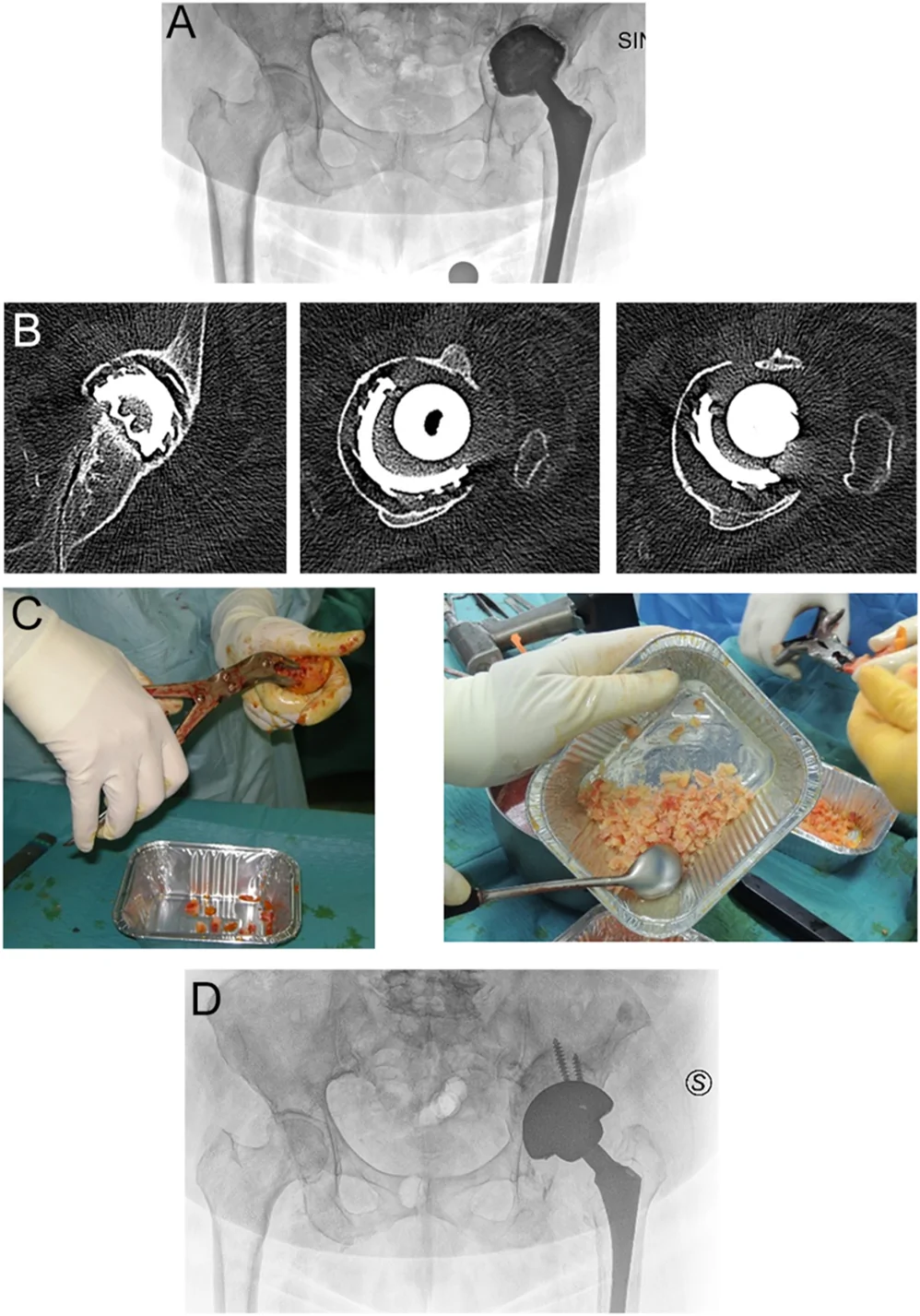
(A) Anteroposterior radiograph of a 77-year-old patient 25 years after a total hip arthroplasty showing aseptic loosening of an uncemented screwed cup. (B) Preoperative CT. (C) Morselized bone allograft. (D) Acetabular bone impaction grafting using an uncemented acetabular cup 2 years after revision surgery.

### Metallic augments and custom-made implants

Continuing advances in surgical technology have led to the development of modern highly porous-coated implants during the two past decades. Modular reconstructions with either tantalum or titanium augments around the acetabulum are considered essential in the modern armamentarium for acetabular revision surgery (Fig. 6). Not only excellent bone fixation but also the association with other reconstruction techniques that use cages, uncemented cups and bone impaction grafting with cemented cups resulted in outstanding results with low re-revision rates and complications in these challenging cases (36). In a recent report, Braunstein *et al.* analysed the mid- and long-term results in 104 patients who underwent revision THA using a porous metal augment construct to manage extensive acetabular bone loss and reported a 10-year survival rate for aseptic loosening of 90.4% (37). The authors reported a 32.7% complication rate, of which 20.2% required further surgeries, and emphasized that the incidence of revision surgeries was higher for both all-cause and aseptic loosening within the flying buttress configuration; however, these results can be considered acceptable in this complex clinical situation.

**Figure 6:**
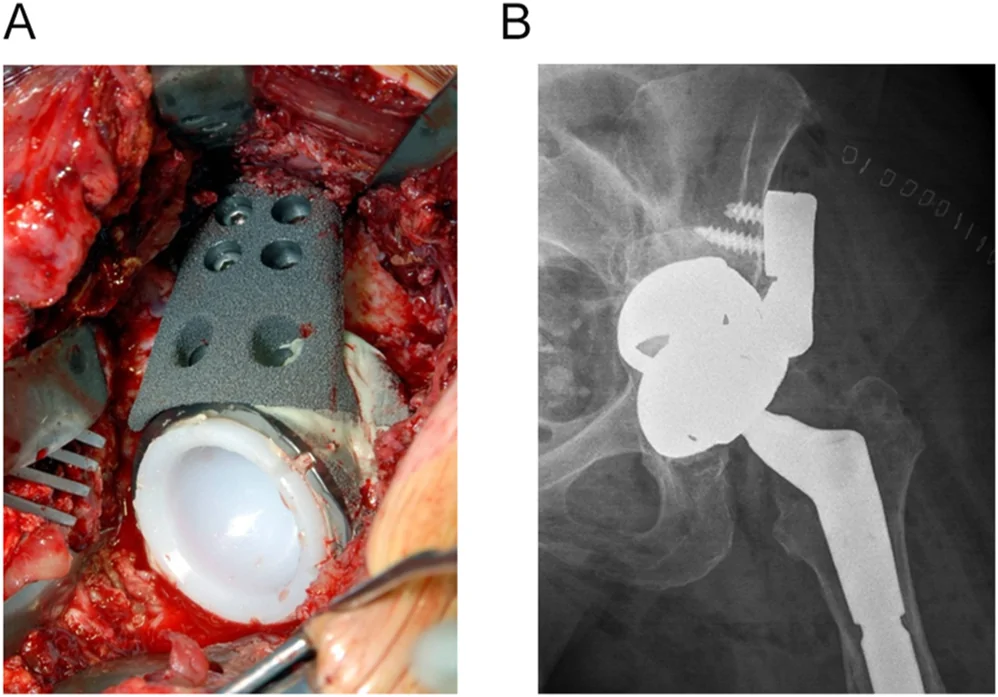
(A) Intraoperative picture showing acetabular revision reconstruction with a highly porous-coated uncemented cup and a lateral trabecular metal augment. Cement between both metallic surfaces can be noted. (B) One-year postoperative radiograph.

Finally, custom-made implants based on 3D CT reconstruction are more commonly used and recommended. Although cost-effectiveness is still undetermined, early results of these implants are promising and improving with the experience of both the surgeons and the engineers that need a strong collaboration for optimizing the results (38). Easier screw placement around the lateral side of the ilium and the pubis and the possibility of increasing stability around the ischium are the most important advantages (Fig. 7). On the contrary, the intraoperative bone loss that occurred during surgery may affect the adaptability of the planned implant. Despite these disadvantages, cemented or modular PE liners and dual-mobility cups can partially solve the problem of mispositioning and prevent from postoperative instability. Furthermore, although new medical device regulations have to confirm the quality and safety of the interface between the coating surface of the implant and the host bone, advanced imaging investigations assessing this concern have shown excellent results (39). Recently, a single-surgeon clinical series including 30 patients who underwent a custom-made, 3D-printed titanium implant with a dual-mobility PE liner, with Paprosky IIIB defects, of those 4 hips had pelvic discontinuity, reported durable fixation at a minimum follow-up of five years (40). Interestingly, they observed that partial postoperative implant–bone contact area showed progressive seating accompanied by osseointegration. Despite this promising bone fixation, there were two dislocations – one transient sciatic nerve palsy and one re-revision due to infection – so the number of complications remains high. Future studies will address issues about costs and manufacturing timing. Nevertheless, it must be pointed out that the indication for such expensive and time-consuming production of these implants is restricted to severe uncontained acetabular bone loss, with or without pelvic discontinuity, failure of previous complex reconstruction or cages (41).

**Figure 7:**
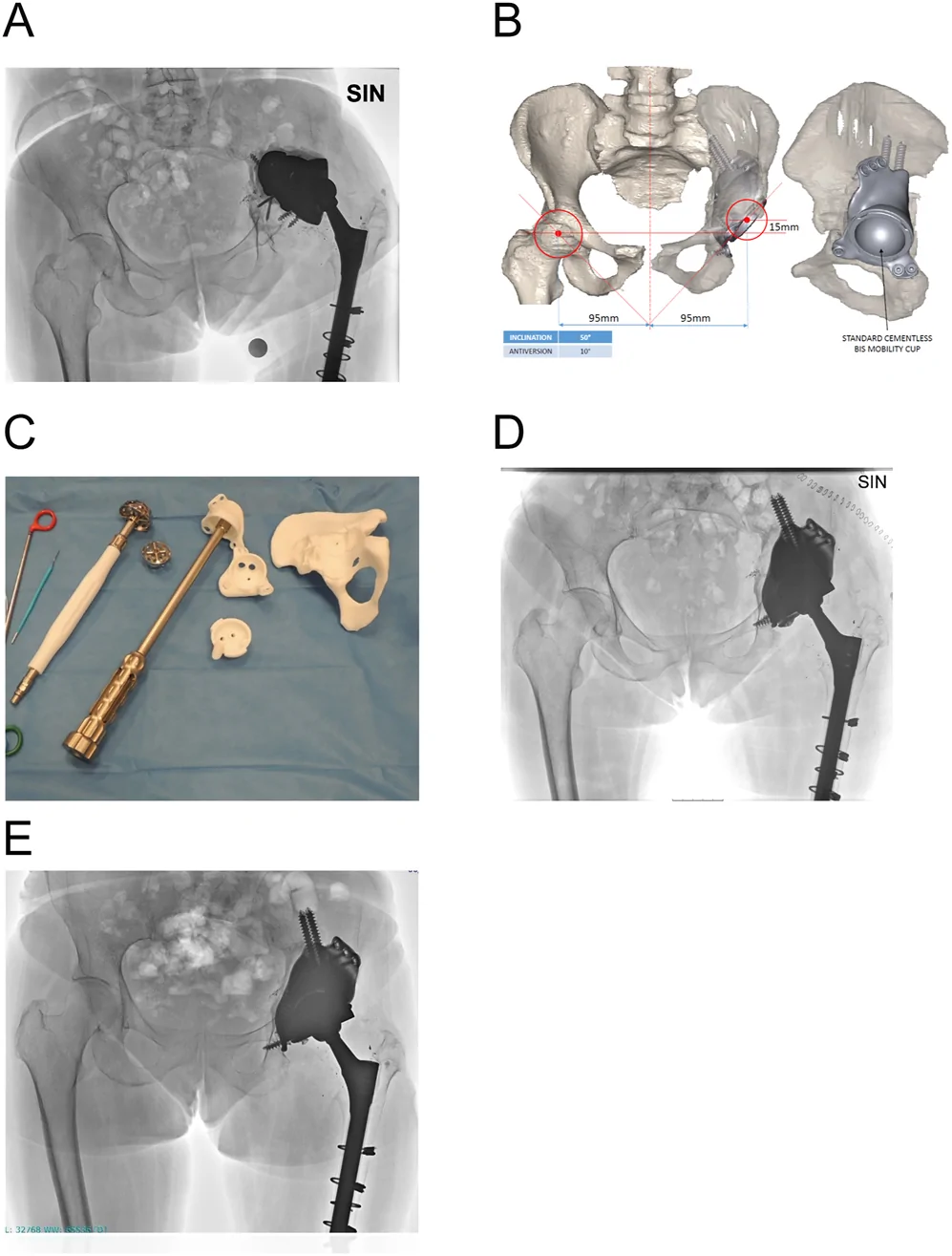
(A) Preoperative radiograph of a 65-year-old patient showing failure of augments in multiple revisions for post-traumatic acetabulum arthritis. (B) Preoperative planning for custom-made acetabular implant. (C) 3D printing biomodel showing instrumentation, acetabular component and femoral bone defect on the acetabular side. (D) Immediate postoperative radiograph. (E) Two-year postoperative radiograph.

### Pelvic discontinuity

The most challenging situation during reconstruction is the existence of pelvic discontinuity. Proximal hemipelvis is not united to distal hemipelvis in an acute or chronic non-union (42). Although this is rare, there is an increasing rate during the past years due to longer life expectancy and the popularity of one-stage surgery performing osteosynthesis and THA in complex acetabular fractures. It is essential to identify whether both hemipelvises are stable using conventional and advanced imaging techniques. Large bone defects may be misunderstood, particularly those with the interruption of the Kohler’s line (Paprosky IIIB), and pelvic discontinuity is frequently overdiagnosed. On the contrary, periacetabular column fractures may be underdiagnosed leading to THA failure if both hemipelvises are not fixed. As Berry *et al.* reported, a visible transverse pelvic fracture with medial offset of the inferior part of the pelvis in relation to the superior part and asymmetry of the obturator ring, indicating rotation of the hemipelvis, are the diagnostic criteria to assess on the AP pelvic views (43).

After identifying whether periprosthetic pelvic discontinuity is acute or chronic, the surgeon must apply the basic principles for fixing both hemipelvises. In most acute lesions, internal fixation using a plate onto the posterior column is the preferred choice. Moreover, morselized bone allografting can be useful to provide some bone stock using impactors manually to increase stability (44). Although modern modular trabecular metal acetabular components are commonly used, an additional reconstruction plate can provide reliable results (42). Similarly, cup-cage reconstruction is effective in cases with both severe defects and associated pelvic discontinuity. Hipfl *et al.* reported no failures due to aseptic loosening in a series of 35 hips, including 24 hips with pelvic discontinuity, at a mean follow-up of 4 years (45). Finally, newer custom-made implants are providing promising results in these cases, although manufacturing issues are yet to be confirmed (40).

## Conclusion

Although wear and osteolysis are less frequently found in hip revision surgery, acetabular reconstruction is still challenging. Intraoperative bone defect determines surgery, so comprehensive knowledge of the implants and orthopaedic bone implant removal armamentarium is a prerequisite before performing the procedure. Furthermore, complications from prior surgeries and all details related to acetabular components and bearings, such as screws, modular inserts or hard-on-hard couples, are to be known.

Modern CT imaging and 3D biomodels are useful to plan cases. Interestingly, future directions to multidisciplinary approaches can improve the outcomes of this difficult surgery. After determining whether an acetabular component can be inserted with a three-point fixation technique into the host bone, alternate methods should be anticipated if this cannot be achieved intraoperatively. Both bone allograft and metallic augments provide reliable results when they are used in combination with cemented or uncemented components (Fig. 8 and Table 1). Future studies focused on basic and clinical research on custom-made implants are yet to be developed in an easy-to-use and meaningful way and on long-term results of such expensive devices. Patient care in this challenging topic is improving, but increasing life expectancy is leading to more surgeries to be performed.

**Figure 8:**
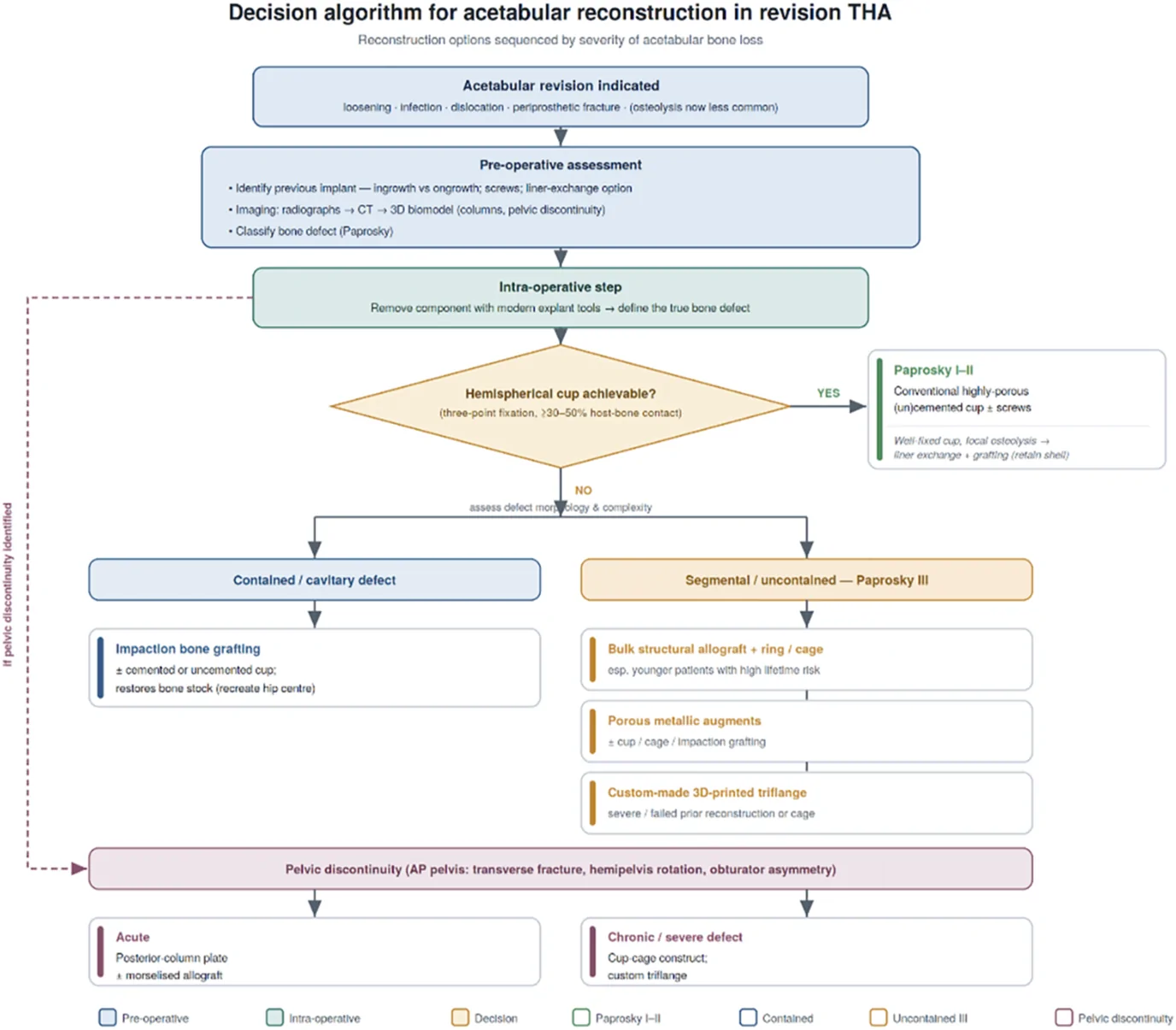
Decision algorithm.

**Table 1:** Reconstruction options for acetabular revision surgery according to the severity of acetabular bone loss. Options are presented in a progressive sequence according to the severity of acetabular bone loss (Paprosky classification).

Bone defect/clinical scenario	Reconstruction option	Key advantages	Limitations	Representative outcomes	References
Paprosky I–II – contained, minor-to-moderate loss; ≥30–50% host–bone contact allowing three-point fixation	Hemispherical highly porous (un)cemented cup ± screws; liner/cement exchange	Simple; reliable biological fixation; preserves bone	Requires sufficient host–bone contact for three-point fixation	Durable long-term fixation in most small/moderate defects	22, 25, 26
Well-fixed cup with focal osteolysis/PE wear; acceptable position; replaceable liner	Retain cup + isolated liner exchange and bone grafting (Maloney)	Avoids removing a well-fixed shell; minimizes iatrogenic bone loss	Infrequent indication; depends on acceptable cup orientation	—	23
Moderate-to-large contained (cavitary) defects	Impaction bone grafting (morselized allograft) ± cemented or uncemented cup	Restores bone stock; recreates near-anatomical hip centre; smaller defect at any re-revision	Technically demanding; graft preparation and vigorous impaction are key steps	Long-term success; combined with cages/metal-mesh for severe defects, including pelvic discontinuity	30, 31, 32, 33, 34, 35
Paprosky III – large segmental/uncontained defects (especially younger patients)	Bulk structural allograft + reinforcement ring/cage (e.g. Kerboull Cross)	Restores bone stock; useful in young patients with high lifetime revision risk	Graft resorption in the weight-bearing zone if not protected by a cage	Gibon: 37 Paprosky III hips – 10-year survival: 95.3% (aseptic loosening) and 76.5% (radiological loosening)	27, 28, 29
Paprosky III – segmental/uncontained defects	Porous metal augments (tantalum/titanium) ± cup, cage or impaction grafting	Excellent fixation; modular; combinable with other techniques	Long-term data and cost-effectiveness debated; non-trivial complication rate	Braunstein: 104 patients – 10-year aseptic-loosening survival: 90.4%; 32.7% complications, 20.2% reoperation	36, 37
Severe uncontained defects/pelvic discontinuity/failed prior reconstruction or cage	Custom-made 3D-printed (triflange) implant ± dual-mobility or cemented liner	Patient-specific; eases ilium/pubis screw placement and ischial stability; DM liner mitigates malposition	Cost and manufacturing time undetermined; intra-op bone loss may compromise fit; high complication rate	Single-surgeon series: 30 Paprosky IIIB hips – durable fixation at ≥5 years, but 2 dislocations – 1 sciatic palsy, 1 re-revision	38, 40, 41
Pelvic discontinuity (acute or chronic)	Posterior column plate ± morselized allograft (acute); cup-cage; custom triflange	Restores hemipelvis continuity; cup-cage reliable for combined severe defect + discontinuity	Over-/underdiagnosis on imaging; chronic non-union most challenging	Hipfl (cup-cage): 35 hips (24 with discontinuity) – no aseptic loosening failures at mean 4 years	42, 43, 44, 45

PE, polyethylene; DM, dual-mobility; aseptic loosening refers to survival free of aseptic loosening.

## ICMJE Statement of Interest

The authors declare that there is no conflict of interest that could be perceived as prejudicing the impartiality of the research reported.

## Funding Statement

This research did not receive any specific grant from any funding agency in the public, commercial or not-for-profit sector.

## Author Contribution Statement

All authors drafted the manuscript, read and revised it critically and approved the final version of the same.
